# Comparing enhanced versus standard Diabetes Prevention Program among indigenous adults in an urban setting: a randomized controlled trial

**DOI:** 10.1186/s12889-020-8250-7

**Published:** 2020-01-30

**Authors:** Lisa G. Rosas, Jan J. Vasquez, Haley K. Hedlin, Fei Fei Qin, Nan Lv, Lan Xiao, Adrian Kendrick, Dawn Atencio, Randall S. Stafford

**Affiliations:** 1 Department of Epidemiology and Population Health, Stanford University, 1701 Page Mill Road, CA, Palo Alto, CA 94304 USA; 2 Department of Medicine, Stanford University, Palo Alto, CA USA; 3 Institute of Health Research and Policy, University of Illinois at Chicago, Chicago, IL USA; 4 American Indian Community Action Board, San Jose, CA USA; 5 Office of Community Engagement, Stanford University, Palo Alto, CA USA

**Keywords:** American Indian and Alaskan natives, Diabetes Prevention Program, Historical trauma, Randomized controlled trial, Body mass index, Metabolic syndrome, Quality of life

## Abstract

**Background:**

Indigenous people in the United States are at high risk for diabetes. Psychosocial stressors like historical trauma may impede success in diabetes prevention programs.

**Methods:**

A comparative effectiveness trial compared a culturally tailored diabetes prevention program (standard group) with an enhanced one that addressed psychosocial stressors (enhanced group) in 2015 to 2017. Participants were 207 Indigenous adults with a body mass index (BMI) of ≥30 and one additional criterion of metabolic syndrome, and were randomized to the standard or enhanced group. Both groups received a culturally tailored behavioral diabetes prevention program. Strategies to address psychosocial stressors were provided to the enhanced group only. Change in BMI over 12 months was the primary outcome. Secondary outcomes included change in quality of life, and clinical, behavioral, and psychosocial measures at 6 and 12 months.

**Results:**

The two groups did not significantly differ in BMI change at 12 months. The two groups also did not differ in any secondary outcomes at 6 or 12 months, with the exception of unhealthy food consumption; the standard group reported a larger mean decrease (95% CI) in consumption of unhealthy food compared with the enhanced group (− 4.6 [− 6.8, − 2.5] vs. -0.7 [− 2.9, 1.4], *p* = 0.01). At 6 months, significant improvements in weight and the physical component of the quality of life measure were observed for both groups compared with their baseline level. Compared with baseline, at 12 months, the standard group showed significant improvement in BMI (mean [95% CI], − 0.5 [− 1.0, − 0.1]) and the enhanced group showed significant improvement in the physical component of the quality of life (2.9 [0.7, 5.2]).

**Conclusions:**

Adding strategies to address psychosocial barriers to a culturally tailored diabetes prevention program was not successful for improving weight loss among urban Indigenous adults.

**Trial Registration:**

(if applicable): NCT02266576. Registered October 17, 2014 on clinicaltrials.gov. The trial was prospectively registered.

## Background

In the United States, American Indian and Alaskan Natives (AIANs) have a disproportionately high prevalence of obesity and diabetes. Self-reported 2016 national data indicate that the prevalence of obesity was 39% and diabetes was 16% among AIANs compared with 29% and 8% in non-Hispanic whites, respectively [1]. Diabetes can be prevented through proven behavioral lifestyle interventions. The 2002 landmark Diabetes Prevention Program (DPP) trial demonstrated that a lifestyle intervention aimed at modest weight loss (5-10% of initial weight) and moderate-to-vigorous physical activity reduced the development of diabetes by 58% over a 3-year period compared with control [2, 3].. Under the well-controlled conditions of the clinical trial, the lifestyle intervention was effective across diverse racial/ethnic groups including AIANs [4]. However, the effectiveness of lifestyle interventions in primary care and community-based settings among racial/ethnic minorities and low SES populations such as AIANs remains a challenge.

For AIAN communities, psychosocial stressors may both increase risk for diabetes as well as hinder preventive efforts such as lifestyle interventions. One such stressor is historical trauma, in which past abuses such as forced removal from ancestral lands and purposeful disintegration of culture through policies such as boarding schools and urban relocation are passed from one generation to another and are found to be inter-generationally cumulative resulting in compounding health effects across generations [5–7]. Greater historical trauma has been found to be associated with increased risk for psychological distress, [8, 9] perceived discrimination, [10] smoking, [11] substance abuse, [12] and contemporaneous trauma such as sexual assault [13]. This proliferation of psychosocial stressors may lead to dysmetabolism and obesity, such as through dysregulation of the hypothalamic–pituitary–adrenocortical axis [14]. Additionally, such psychosocial stressors may impede successful implementation of diabetes prevention [15–20].

Identifying successful and innovative strategies to address psychosocial stressors, such as historical trauma is particularly salient for AIANs who have a high risk for diabetes. This study used a community-based participatory research approach to compare an enhanced DPP for AIAN adults that incorporated culturally sensitive strategies to address psychosocial stressors to a standard DPP in a comparative effectiveness trial. We hypothesized that an enhanced strategy would improve Body Mass Index (BMI) at 12 months as compared with the standard DPP.

## Methods

The Institutional Review Boards of Stanford University approved the entire study protocol. All participants provided written informed consent. The study trial protocol was published previously [21].

### Community engagement

A community-university partnership known as Pathways to American Indian and Alaska Native Wellness used a community-based participatory research approach to design and conduct this comparative effectiveness trial in 2015 to 2017. A community advisory board of community members and leaders known as the American Indian Community Action Board (AICAB) was the central governing body of the partnership and was integrally involved in all phases of the study including conceptualization, implementation, and analysis of results.

### Study participants and setting

This study was conducted in Santa Clara County, CA which is home to a diverse urban Indigenous population including AIANs as well as those who are Indigenous to Mexico and other Latin American counties. Thus, Indigenous adult men and women aged 18 years and older were recruited in 5 cohorts through community outreach at local clinics, community-based organizations (e.g., Intertribal Friendship House), retail locations (e.g., pharmacies), and schools. Inclusion criteria included: self-identification as Indigenous to the US or the Americas (North, Central, and South America; referred to as “Indigenous” from hereon), a BMI between 30 and 55 kg/m^2^, no diagnosis of Type 2 Diabetes, and at least one other criterion for metabolic syndrome: [1] Triglycerides: >150 mg/dL [2]; Reduced High-density lipoprotein cholesterol: <40 mg/dL (men); <50 mb/dL (women) [3]; Blood pressure: >130/80 mmHg or current treatment with antihypertensives [4]; Fasting glucose: 100-125 mg/dL. These inclusion criteria were chosen to identify a population who were at risk of developing diabetes and could potentially benefit from the intervention. People with significant psychiatric disorders requiring atypical antipsychotics or multiple medications or medical comorbidities (e.g., uncontrolled metabolic disorders, unstable heart disease, heart failure, and ongoing substance abuse) were excluded. Additional exclusions were to protect participant safety (e.g., pregnancy) and prevent loss to follow-up (e.g., planned relocation). There was no gender bias in the selection of participants.

### Treatment groups

#### Standard intervention group

The Standard intervention was based on the Special Diabetes Program for Indians (SDPI), a group-based adaptation of the original one-on-one DPP lifestyle intervention whose effectiveness has been previously reported [22]. The SDPI modified the original DPP intervention by offering group sessions, adapting examples and graphics to be appealing to AIAN adults, and providing participant incentives such as running shoes. The intervention is grounded in Social Cognitive Theory [23] and the Transtheoretical Model of Behavior Change [24]. The primary goals of the SDPI intervention are loss of at least 5% of baseline weight and 150 min of moderate physical activity per week by 6 months. Although the original DPP trial targeted 7% weight loss, 5% weight loss has been found to be sufficient for prevention of chronic disease and is commonly accepted as the goal [25]. The intervention was delivered by a trained lifestyle coach over 16 weekly group sessions covering information on moderate calorie restriction, physical activity, and proven behavioral strategies.

#### Enhanced intervention group

Participants randomized to the enhanced intervention participated in the standard intervention and were offered the opportunity to participate in three different enhancements that were developed and pilot tested by the AICAB to address psychosocial stressors, such as historical trauma. Based on recommendations from the AICAB, participants worked with their lifestyle coach to determine which enhancements were appropriate for them. The lifestyle coach for the enhanced intervention group was trained in DPP and in the three added psychosocial support components. If needed, the coach could consult with a licensed clinical social worker for guidance. The enhancements included:

1. Talking circles were added to sessions 3, 8, and 15. Talking circles are a traditional method of group communication where AIAN community members come together to share information, provide social support, and solve community issues [26]. Talking circles have been successfully used as an intervention strategy for health issues ranging from cervical cancer screening to diabetes management [26–28].
2. A modified *Photovoice* activity was incorporated into sessions 3, 8, 14, and 15. Photovoice can be used to highlight for participants the multi-level factors, such as food scarcity, social influences, and government policies that shape diet and physical activity. The goals of the modified *Photovoice* were to engage participants to record and reflect on their strengths and weaknesses regarding making lifestyle changes [29, 30].
3. Digital story sessions were offered as an option outside of the regular sessions. Digital stories are short, first-person narratives presented using either traditional or social media formats. The participatory process of developing and sharing digital stories can deeply affect both the person who develops their story as well as viewers, and can contribute to modifying personal behaviors [31].

### Randomization and blinding

Eligible participants were randomized in a 1:1 ratio to receive the standard or enhanced DPP. Participants were randomized in blocks to keep the size of the treatment groups similar. The size of each block was randomly selected to be either 2 or 4. To ensure an equal number of males and females in each intervention arm, we stratified randomization by gender. Treatment was identifiable to participants and the lifestyle coaches by design, but masking of the investigators, Data and Safety Monitoring Board, outcome assessors, and the statistician performing the data analysis were enforced.

### Outcome measures

Participants were assessed at baseline, 6 months, and 12 months. All outcome assessors were trained to perform the measurements and interviews per standardized protocols and procedures.Our primary outcome was BMI at 12 months. Weight and height were assessed according to standard protocols [32]. Secondary outcomes included quality of life, health behaviors (i.e., diet and physical activity), clinical factors (i.e., waist circumference, blood pressures, fasting glucose, high-density lipoprotein cholesterol [HDL], low-density lipoprotein cholesterol [LDL], triglyceride), and psychosocial factors (i.e., depression and empowerment). The quality of life outcome was emphasized in the analysis based on the AICAB’s interest in this patient-centered outcome. The SF-12 was used to measure quality of life, which has been used in other studies with AIAN adults [33, 34]. Dietary data were collected using a food frequency questionnaire modified to incorporate culturally-relevant food choices (e.g. corn tortillas and frybread) [35]. Food items were scored on a scale of 1 to 6, with 6 corresponding to the greatest frequency of consumption. Food items on the food frequency questionnaire were categorized as “healthy,” “unhealthy,” and “undetermined” based on classifications previously determined by Teuful-Shone et al. [35] Healthy and unhealthy food scores were obtained by dividing the sum of food items in each category into tertiles, with the third tertile indicating the highest consumption frequency. Undetermined food scores were not used for analysis. Physical activity was measured using the Women’s Health Initiative physical activity questionnaire with modifications to reflect the recall time [36]. Trained staff conducted anthropometric and blood pressure measurements [32, 37]. Measurements of fasting glucose and lipid levels were obtained from point-of-care testing (Cholestech) to minimize patient burden, maximize access, and provide immediate results. Depression was assessed using the Center for Epidemiological Studies-Depression (CES-D) scale [38]. Empowerment was measured using the Growth and Empowerment Measure, consisting of a 14-item Emotional Empowerment Scale and a 12-item Scenarios scale, designed to assess change in dimensions of empowerment [39].

Additional participant characteristics collected included sociodemographic characteristics, food security, alcohol consumption, sleep disturbance and impairment, and posttraumatic stress disorder (PTSD). Sociodemographic characteristics included age, sex, race/ethnicity, income, and educational attainment. Food security was measured using the 6-item Short Form of the US Household Food Security Survey and participants were categorized as having “very low food security, “low food security,” or “high food security” [40]. Alcohol consumption was assessed using the AUDIT-C [41] and sleep habits and quality were assessed using the PROMIS questionnaire [42]. PTSD was measured using the 17-item PTSD checklist – Civilian Version [43]. Sociodemographic characteristics and food security was measured at baseline only.

### Statistical analysis

Mean and standard deviation (SD) for continuous variables and N and percentage for categorical variables were used for descriptive statistics and session attendances.

Four classes of analyses were performed: 1) between group differences on primary and secondary outcomes (the primary analysis); 2) within group difference on primary and secondary outcomes; 3) effect modification analysis for primary outcome; and 4) session attendance and its association with the primary outcome. Intention-to-treat analyses of between-treatment differences in primary and secondary outcomes tested for treatment-by-time interactions in repeated-measures mixed-effects linear or generalized linear models with a logit link for binary outcomes (i.e., 5% weight loss at 6 and 12 months follow-ups). The fixed effects of each model consisted of gender, treatment, time point (baseline, 6, or 12 months), and treatment-by-time interaction. The random effects accounted for repeated measures with an unstructured covariance matrix and clustering of patients within cohorts. The model is described in more detail in the trial protocol paper.^12^ Missing data were handled directly through maximum-likelihood estimation in mixed modelling. We also verified the mixed model-based results with multiple imputation analysis. Effect modification was investigated using mixed effects linear regression by including an interaction term of treatment and the hypothesized effect modifier [44]. Potential effect modifiers included Indigenous ancestry (i.e., US Indigenous people vs. non-US Indigenous people), income, depression, and food insecurity. Because the Indigenous population in the local area is diverse and those from different ancestral backgrounds have a different experience of historical trauma, Indigenous ancestry was identified as a potential effect modifier. Similarly, depression and food insecurity were identified as other psychosocial stressors that may impact the effectiveness of the intervention. The adherence dose effects combining both groups were examined using the same mixed effects linear model except that treatment was replaced by the number of attended sessions.

All analyses were conducted using SAS, version 9.4 (SAS Institute Inc., Cary, North Carolina). The targeted sample size of 102 participants in each group was designed to provide 80% power to detect an effect size of 0.45 at 5% α (2-sided) in the primary outcome between enhanced and standard groups assuming up to a 20% loss to follow-up at 12 months.

## Results

### Study participants and baseline characteristics

**Fig. 1:**
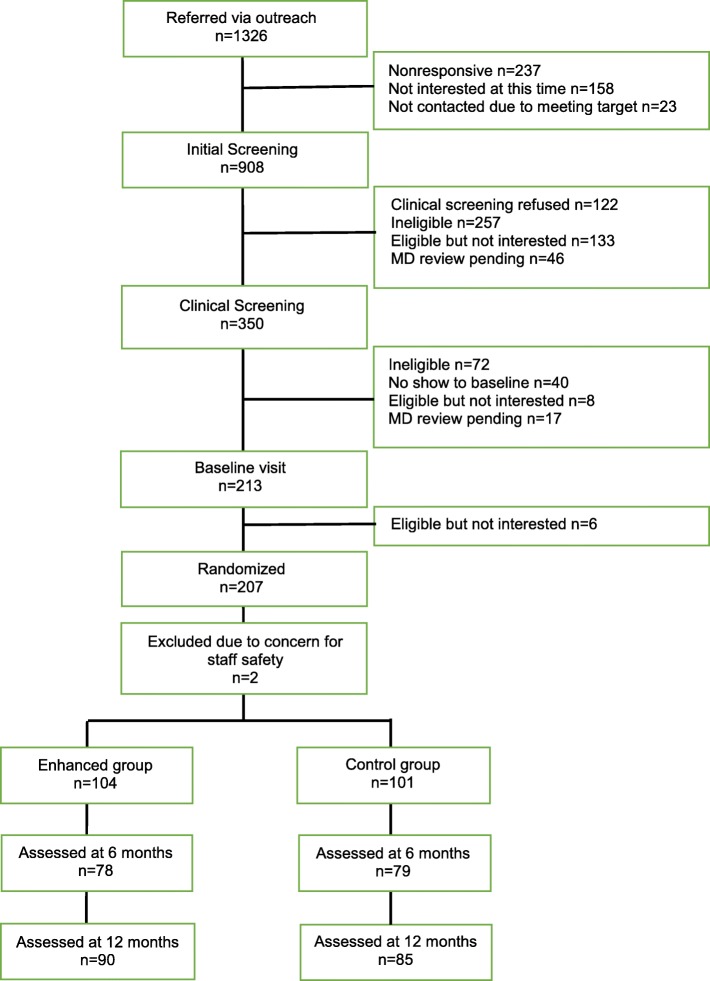
Consort chart

Of the 1326 potential participants referred through community-based outreach, 908 completed initial screening, and 418 people did not complete the screening. Of the 418 who did not complete the screening, staff attempted to contact 237 people but they were nonresponsive, 158 people were reached but were not ready to commit at that time and asked to be contacted in the future, and 23 people were not called because the enrollment target was met. Of the 908 who completed initial screening, 379 were not eligible or declined participation at that stage, 133 were eligible but not interested, 46 needed physician approval, and 350 completed clinical screening. Among the 350 who completed clinical screening, 278 were eligible or needed physician approval, of whom 213 completed baseline visit. This process yielded the sample size of 207 eligible and consenting participants. Two participants were excluded post randomization due to safety concerns for the study staff. Of the 207 randomized participants, 157 (76%) were assessed at 6 months and 175 (85%) at 12 months (Fig. 1).

Participants were middle-aged (mean [SD], 52.0 [13.3]), mostly female (78.7%), and obese (BMI, 37.5 [6.6] for men and 37.2 [6.0] for women); with Indigenous ancestry from multiple regions (Table 1). At baseline, 14% of participants were hazardous drinkers. Participants had a sleep disturbance T-score of 52.7 (3.8) and a sleep-related impairment T-score of 53.6 (7.4). In addition, 34% of participants reported symptoms consistent with depression (CES-D ≥ 16). Their mean systolic blood pressure was 123.6 (SD 17.1) mm Hg, diastolic blood pressure 77.9 (11.9) mm Hg, fasting glucose 100.7 (10.7) mg/dL, HDL 49.9 (14.7) mg/dL, LDL 101.5 (29.1) mg/dL, and triglyceride 142.2 (82.1) mg/dL.

**Table 1:** Baseline participant characteristics overall and by arm in San Jose, California (*n* = 207)*

	Total	Enhanced intervention	Standard intervention
	*n* = 207	*n* = 105	*n* = 102
Sociodemographic characteristics			
Age, years, mean ± SD	52.0 ± 13.3	52.1 ± 13.8	51.9 ± 12.8
Female	78.7%	79.0%	78.4%
Indigenous Person (IP)^a^	97.1%	98.1%	96.1%
Race			
IP US, Canada	45.4%	49.5%	41.2%
IP Mexica, Central America, South America	30.0%	28.6%	31.4%
IP Mixed Ancestry	24.6%	21.9%	27.4%
Ethnicity			
Hispanic	53.6%	54.3%	52.9%
Non-Hispanic	46.4%	45.7%	47.1%
Income, *n* = 205 (103, 102)			
< $20,000	37.5%	35.9%	39.2%
$20,000 - $50,000	35.7%	38.9%	32.3%
$50,000+	26.8%	25.3%	28.5%
Education			
< High school graduate (0–11 years)	13.5%	16.2%	10.8%
High school graduate (12 years)	20.8%	19.0%	22.5%
Some college (1–3 years)	44.0%	44.8%	43.1%
College graduate (4 or more years)	12.6%	11.4%	13.7%
Graduate degree	9.2%	8.6%	9.8%
Behavioral characteristics			
Healthy Food Score tertiles			
9–21	35.8%	34.3%	37.3%
22–26	43.5%	44.8%	42.2%
27–32	20.8%	20.9%	20.6%
Unhealthy Food Score tertiles			
14–31	33.3%	36.2%	30.4%
32–39	37.7%	40.9%	34.3%
39–58	29.0%	22.9%	35.3%
Physical Activity, *n* = 194 (98, 96)			
< 500 MET-Minutes/Week	52.1%	54.1%	50.0%
500–1000 MET-Minutes/Week	20.0%	19.4%	20.8%
> 1000 MET-Minutes/Week	27.8%	26.5%	29.2%
Hazardous drinking - AUDIT-C	13.5%	12.4%	14.7%
PROMIS sleep disturbance T score, mean ± SD, *n* = 203 (103, 100)	52.9 ± 9.4	53.1 ± 8.6	52.7 ± 10.1
PROMIS sleep-related impairment T score, mean ± SD, *n* = 192 (98, 94)	46.7 ± 7.9	47.0 ± 8.1	46.3 ± 7.7
Clinical characteristics			
BMI (men), kg/m2, mean ± SD	37.5 ± 6.6	39.5 ± 7.8	35.5 ± 4.7
BMI (women), kg/m2, mean ± SD	37.2 ± 6	37.2 ± 6.2	37.1 ± 5.8
Weight (men), lb., mean ± SD	248.8 ± 59	262.9 ± 67.1	234.6 ± 47.1
Weight (women), lb., mean ± SD	204 ± 35.3	206.6 ± 37.9	201.2 ± 32.3
Waist circumference (men), in, mean ± SD	47.8 ± 6.5	49.1 ± 7.3	46.5 ± 5.5
Waist circumference (women), in, mean ± SD, *n* = 160 (82, 78)	46.2 ± 5.4	45.9 ± 5.6	46.5 ± 5.1
SBP, mm Hg, mean ± SD	123.6 ± 17.1	123.4 ± 17.2	123.8 ± 17.1
DBP, mm Hg, mean ± SD	77.9 ± 11.9	78.5 ± 11.9	77.3 ± 11.9
Fasting glucose, mg/dL, mean ± SD	100.7 ± 10.7	101.2 ± 10.9	100.1 ± 10.5
HDL, mg/dL, mean ± SD	49.9 ± 14.7	49.7 ± 15	50.1 ± 14.5
LDL, mg/dL, mean ± SD	101.5 ± 29.1	103.5 ± 31	99.5 ± 27
Triglycerides, mg/dL, mean ± SD	142.2 ± 82.1	134.8 ± 69.4	149.7 ± 93.1
Psychosocial characteristics			
Depression –CES-D, mean ± SD	14 ± 11	13.1 ± 9.8	14.9 ± 12.1
Depressed (CES-D ≥ 16)	34.3%	31.4%	37.3%
PTSD - PCL-C, mean ± SD, *n* = 198 (102, 96)	30.1 ± 12.4	29.5 ± 11.7	30.7 ± 13
Food Security			
Very Low Security	16.4%	17.1%	15.7%
Low Security	25.1%	21.9%	28.4%
High Security	58.5%	61.0%	55.9%
Empowerment			
Emotional empowerment	46.4 ± 9.1	46.3 ± 8.7	46.5 ± 9.6
Inner peace	27.2 ± 5.4	27.2 ± 5.1	27.1 ± 5.8
Self-capacity	11.6 ± 2.7	11.5 ± 2.7	11.7 ± 2.6
Scenarios	60.1 ± 13	59.8 ± 13.1	60.4 ± 12.9
Healing	35.9 ± 7.6	35.9 ± 7.5	35.8 ± 7.7
Connection	24.2 ± 6.4	23.9 ± 6.6	24.6 ± 6.1

Abbreviations: *CES-D* Center for Epidemiological Studies-Depression Scale; *DBP* diastolic blood pressure; *HDL* high-density lipoprotein cholesterol; *LDL* low-density lipoprotein cholesterol; *PTSD – PCL-C* posttraumatic stress disorder Checklist – Civilian Version; *SBP* systolic blood pressure

* *n* = 207 unless otherwise specified as *n* = # of total (# of enhanced intervention, # of standard intervention)

^a^Although the eligibility criteria specified self-identification of having indigenous ancestry, a small number of participants who reported having indigenous ancestry preferred to self-identify as a different race

### Primary and secondary outcomes

At 6 months, mean net change (95% confidence interval [CI]) in BMI from baseline did not differ for participants in the enhanced intervention (− 0.3 [− 0.7, − 0.02]) compared with the standard intervention (− 0.7 [− 1.0, − 0.4]) (*p* = 0.12). At 12 months, mean net change (95% CI) in BMI from baseline did not differ for the enhanced intervention (− 0.3 [− 0.7, 0.2]) compared with the standard intervention group (− 0.5 [− 1.0, − 0.1]) (*p* = 0.39). The mean percent (95% CI) of participants with 5% weight loss did not differ for the enhanced group (10.4% [4.4, 22.7%]) compared with the standard group (20.7% [10.3, 37.1%]) at 6 months [*p* = 0.12) and did not differ between the two groups (18.3% [9.0, 33.5%] vs. 23.7% [12.3, 40.7%]) at 12 months (*p* = 0.48).

The two treatment groups also did not differ significantly in changes in quality of life, behavioral (i.e., diet and physical activity MET minutes), clinical (i.e., waist circumference, blood pressures, fasting glucose, HDL, LDL, triglyceride), and psychosocial secondary outcomes (i.e., depression and empowerment) at 6 and 12 months, except for changes in unhealthy food consumption frequency at 12 months (Table 2). At 12 months, participants in the standard intervention had a larger mean decrease (95% CI) in unhealthy food consumption frequency compared with those in the enhanced intervention (− 4.6 [− 6.8, − 2.5] vs. -0.7 [− 2.9, 1.4]) (*p* = 0.01).

**Table 2:** Estimated means and standard errors/95% confidence intervals for primary and secondary outcomes (*n* = 205)

	Baseline		Change from baseline to 6 months		Change from baseline to 12 months	
	Enhanced	Standard	Enhanced	Standard	Enhanced	Standard
Primary Outcomes						
BMI, kg/m^2^	37.8 ± 0.7	37.0 ± 0.7	− 0.3 (− 0.7,-0.02)	− 0.7 (− 1.0, − 0.4)	− 0.3 (− 0.7, 0.2)	− 0.5 (− 1.0, − 0.1)
Percentage of participants with 5% weight loss	N/A	N/A	10.4 (4.4, 22.7)	20.7 (10.3, 37.1)	18.3 (9.0, 33.5)	23.7 (12.3, 40.7)
Secondary Outcomes						
SF-12						
Physical component	40.2 ± 1.3	41.5 ± 1.3	3.1 (1.0, 5.2)	3.0 (0.9, 5.1)	2.9 (0.7, 5.2)	2.2 (−0.1, 4.5)
Mental component	48.0 ± 1.2	47.1 ± 1.2	1.7 (− 1.0, 4.3)	1.5 (− 1.1, 4.2)	0.3 (− 2.4, 3.0)	2.0 (− 0.7, 4.7)
Behavioral outcomes						
Diet						
Healthy	22.8 ± 0.5	22.6 ± 0.5	0.4 (− 0.7, 1.5)	− 0.6 (− 1.7, 0.5)	−0.4 (− 1.6, 0.9)	0.4 (− 0.9, 1.7)
Unhealthy	35.4 ± 1.0*	37.9 ± 1.0*	− 2.51 (− 4.4, − 0.6)	−4.6 (− 6.5, − 2.8)	− 0.7 (− 2.9, 1.4)*	−4.6 (− 6.8, − 2.5)*
Physical activity MET mins/week	1540.6 ± 150.3	1389.1 ± 149.7	− 23.5 (− 314.8, 267.8)	−94.3 (− 386.2, 197.6)	− 241.6 (− 591.9, 108.7)	144.6 (− 207.6, 496.8)
Clinical outcomes						
Waist circumference, in	47.2 ± 0.7	47.1 ± 0.7	− 2.1 (− 2.8, − 1.4)	− 2.7 (− 3.4, − 2)	− 1.8 (− 2.6, − 1)	−2.5 (− 3.2, − 1.7)
SBP, mm Hg	125.3 ± 1.9	125.8 ± 1.9	−5.5 (− 8.7, − 2.2)	− 2.8 (− 5.9, 0.3)	− 4.1 (− 7.4, − 0.8)	−2.6 (− 5.8, 0.6)
DBP, mm Hg	80.0 ± 1.3	78.8 ± 1.3	−4.4 (− 6.6, − 2.2)	−0.5 (− 2.7, 1.6)	− 1.2 (− 3.6, 1.2)	0.05 (−2.3, 2.4)
Fasting glucose, mg/dL	101.0 ± 1.3	99.5 ± 1.3	1.9 (− 0.7, 4.4)	2.3 (− 0.2, 4.7)	5.0 (1.9, 8.0)	4.9 (1.9, 7.8)
HDL, mg/dL	46.5 ± 1.6	46.9 ± 1.6	0.03 (− 1.9, 1.9)	1.0 (− 0.8, 2.8)	0.7 (− 1.5, 3)	0.7 (− 1.5, 3)
LDL, mg/dL	102.9 ± 3.5	100.2 ± 3.5	1.6 (− 3.9, 7)	−0.5 (− 5.7, 4.7)	4.5 (− 2.9, 11.9)	2.8 (− 4.3, 9.9)
Triglyceride, mg/dL	134.0 ± 8.7	146.9 ± 8.8	13.2 (− 4.2, 30.7)	−2.0 (− 19.0, 15.0)	21.7 (1.8, 41.7)	− 2.9 (− 22.5, 16.6)
Psychosocial outcomes						
CES-D	12.2 ± 1.1	13.9 ± 1.2	0.04 (− 2.2, 2.3)	−2.3 (− 4.6, − 0.1)	−0.1 (− 2.7, 2.6)	−1.2 (− 3.8, 1.5)
Empowerment						
Emotional empowerment	47.1 ± 1.1	46.2 ± 1.1	− 0.4 (− 2.7, 1.9)	0.2 (−2, 2.5)	0.9 (− 1.5, 3.2)	−0.3 (− 2.7, 2)
Inner peace	27.7 ± 0.6	27.1 ± 0.7	−0.6 (− 2, 0.8)	0.2 (−1.1, 1.6)	0.4 (− 1, 1.8)	−0.2 (− 1.6, 1.2)
Self-capacity	11.5 ± 0.4	11.3 ± 0.4	0.1 (− 0.6, 0.8)	−0.2 (− 0.8, 0.5)	0.2 (− 0.6, 0.9)	−0.3 (− 1.1, 0.4)
Scenarios	57.1 ± 1.7	59.9 ± 1.8	−1.9 (− 5.1, 1.3)	−2.3 (− 5.6, 1)	−2.1 (− 5.3, 1.2)	0.2 (− 3.1, 3.5)
Healing	33.5 ± 1.1	35.1 ± 1.1	− 1.4 (− 3.5, 0.6)	−1.6 (− 3.6, 0.5)	−1.9 (− 4, 0.1)	− 0.3 (− 2.4, 1.7)
Connection	23.6 ± 0.8	24.9 ± 0.8	−0.5 (− 2.0, 1.0)	−0.7 (− 2.2, 0.8)	−0.1 (− 1.7, 1.4)	0.5 (−1.1, 2.1)

Abbreviations: *BMI* body mass index; *CES-D* Center for Epidemiological Studies-Depression Scale; *DBP* diastolic blood pressure; *HDL* high-density; lipoprotein cholesterol; *LDL* low-density lipoprotein cholesterol; *N/A* not applied; *SBP* systolic blood pressure; *SF-12* Short Form 12 Health Survey

Boldface indicates between groups statistical significance (*P < 0.05)

**Fig. 2:**
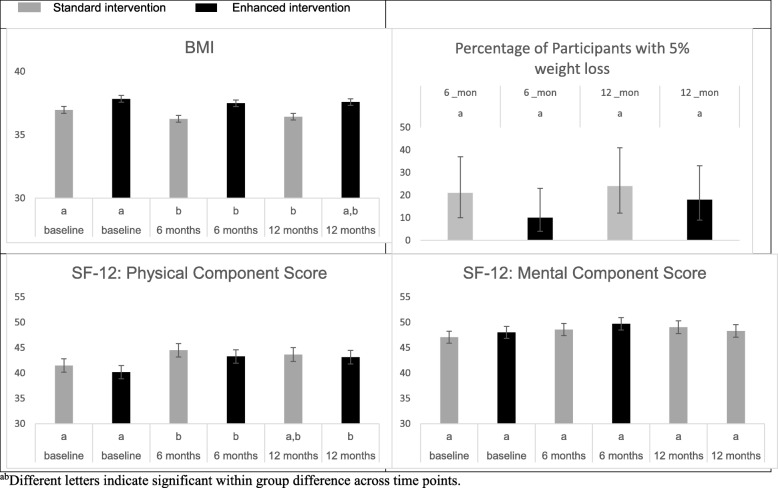
Estimated means and standard errors for BMI and SF-12 by group, ^ab^Different letters indicate significant within group difference across time points

Figure 2 shows within group differences on BMI and SF-12 over time. Both treatment groups had a significantly lower BMI at 6 months compared with baseline (mean [95% CI], − 0.3 [− 0.7, − 0.02], *P* = 0.04 for the enhanced intervention and − 0.7 [− 1.0, − 0.4], *P* < 0.0001 for the standard intervention group); however, only participants in the standard intervention had a statistically significantly lower BMI at 12 months compared with baseline (− 0.5 [− 1.0, − 0.1], *P* = 0.02). The percent (95% CI) of participants with 5% weight loss did not differ between 6 and 12 months within each treatment group. Participants in the enhanced intervention had a significantly higher SF-12 physical component score at both 6 months (3.1 [1.0, 5.2], *P* = 0.004) and 12 months (2.9 [0.7, 5.2], *P* = 0.01) compared with baseline, while participants in the standard intervention only had a significantly higher SF-12 physical component score at 6 months (3.0 [0.9, 5.0], *P* = 0.005). SF-12 mental component score did not change significantly over time within either the enhanced or standard intervention group.

### Effect modification

Effect modification analysis showed that baseline Indigenous ancestry (i.e., US Indigenous people vs. non-US Indigenous people), income, depression, and food insecurity did not modify the intervention effects on the primary outcome.

### Session attendance

Out of a total of 16 weekly sessions, mean (SD) number of sessions attended was not significantly different between the enhanced intervention group (9.5 [5.7]) and the standard intervention group (9.0 [5.3]). Of the enhanced intervention participants, 79% completed at least 4, 62% completed at least 8, and 46% completed at least 12 sessions. Of the standard intervention participants, 80% completed at least 4, 62% completed at least 8, and 38% completed at least 12 sessions. In the enhanced intervention group, 21 (20%) participants did not attend any Photovoice sessions, 19 (18%) participants attended 1 out of a total of 4 sessions, 13 (13%) attended 2, 13 (13%) attended 3, and 37 (36%) attended all 4 sessions. Fifty (49%) participants did not attend any Talking circles, 19 (18%) participants attended 1 out of a total of 3 Talking circles, 15 (15%) attended 2, and 19 (18%) attended all 3 Talking circles. Only one participant attended both digital storytelling sessions offered; the rest did not attend any digital storytelling session.

Repeated-measures mixed-effects linear models among all participants combined indicated that higher adherence was significantly associated with a greater decrease in BMI. For BMI, the mean change (95% CI) with each additional session attended was − 0.06 (− 0.10, − 0.01) (*P* = 0.01) at 6 months and − 0.07 (− 0.13, − 0.01) (*P* = 0.03) at 12 months.

## Discussion

This comparative effectiveness trial compared two approaches to diabetes prevention among Indigenous adults at high risk for developing diabetes in an urban area. Contrary to our hypothesis, the standard and enhanced interventions did not significantly differ in change of BMI. Among secondary outcomes, no differences were noted except for dietary intake where the standard group reported a larger decrease in unhealthy food consumption compared with the enhanced group. However, some participants in both groups were successful in reducing their BMI and improving their quality of life, which has implications for future research directions.

There are several possible explanations for why the standard and enhanced groups did not differ on the primary and secondary outcomes. First, it is possible that it is not necessary to address historical trauma in the context of diabetes prevention for urban Indigenous adults. Documentation among AIAN residing on reservations suggests that historical trauma as measured by the historical loss scale and the historical loss associated symptoms scale is common [45]. For example, among 143 AIAN adults recruited from two reservations in the American Midwest, the Historical Losses most commonly thought about weekly, daily, or several times a day included “Loss of respect by our children and grandchildren for elders” (65%), “The losses from the effects of alcoholism on our people” (64%), “Losing our traditional spiritual ways” (55%), “Loss of our people through early death” (55%), and “Loss of respect by our children for traditional ways” (53%). In contrast, participants in this trial reported experiencing historical trauma less often. Among participants who reported ancestry from the US and Canada, the top five historical losses thought about at least weekly included “Losses from the effects of drugs on our people” (31%), “Losses from the effects of alcoholism on our people” (29%), “Loss of respect by our children and grandchildren for elders” (29%), “Losing our culture” (24%), and “Loss of respect by our children for traditional ways” (23%). Among participants who reported ancestry from Mexico, Central America, and South America, reports of experiencing historical trauma were less frequent: “Loss of respect by our children and grandchildren for elders” (20%), “Loss of respect by our children for traditional ways” (19%), “The loss of our land due to the Spanish conquest or colonization” (14%), “The losses from the effects of alcoholism on our people” (12%), and “The losses from the effects of drugs on our people” (12%). Given that historical trauma is less common among urban AIAN adults compared with those residing on reservations, it is possible that addressing this psychosocial barrier for the purposes of augmenting the effectiveness of diabetes prevention is not needed.

Second, addressing additional barriers other than historical trauma is potentially more important. Both intervention groups addressed numerous barriers that Indigenous populations commonly face for successful diabetes prevention such as transportation, competing priorities of work and caretaking, and lack of safe places for physical activity. Strategies to address these barriers were for participants in both groups and included hosting the intervention at convenient times and in a location accessible by public transport, providing membership to a gym (either on site or in a location convenient to the participant) and other incentives such as a healthy meal during the class and comfortable athletic shoes. It is possible that addressing these barriers was sufficient for this urban population and that additional strategies to address historical trauma were not needed.

Third, enhancements developed to address psychosocial stressors may not have been effective or engagement in the enhancements may not have been sufficient to be effective. The enhancements were primarily developed to address historical trauma, which is a complex issue that may require more in-depth or long-term intervention than is feasible in the context of a diabetes prevention intervention [5, 46, 47]. Additionally, other psychosocial concerns may be important to address in addition to historical trauma to promote effectiveness. In the SDPI, participants with psychological distress and negative family support lost less weight than those that did not face these barriers [48]. Alternatively, it is possible that participants did not receive a sufficient dose of the enhancements. Intervention staff and AICAB members put forth considerable effort to promote participation, however approximately one-third (36%) attended all four photovoice sessions and one fifth (18%) attended all three talking circles. Only one person completed the digital storytelling.

Despite the fact that the groups did not differ according to the primary outcome, participants in both groups made significant improvements in BMI and quality of life compared with their baseline levels. Overall 18% of participants in the enhanced group and 24% in the standard group lost at least 5% weight at 12 months. In addition, our study found that increased attendance was associated with greater weight loss at both 6 and 12 months. This is consistent with other studies that have documented the benefit of increasing the number of sessions attended [49, 50]. The importance of this finding is reflected in policies from the Centers for Disease Control and Prevention Diabetes Prevention Program that provides recognition to DPP providers. To achieve recognition, the CDC requires that at least 60% of participants attend at least 9 sessions during months 1–6 and at least 60% of participants attend at least 3 sessions in months 7–12 [51]. Based on these findings, future research aimed at increasing effectiveness and session attendance is warranted. These efforts could include additional strategies that focus on addressing social determinants of health and/or refinement of the target population. In the recent Kerala DPP trial, a low-cost community-based peer-support DPP intervention resulted in a nonsignificant reduction in diabetes incidence at 24 months; however, the intervention was effective in the subgroup with impaired glucose tolerance and ineffective in the subgroup with impaired fasting glucose. (52) We do not have information on the proportion of participants with impaired glucose tolerance versus fasting glucose. However, a higher proportion of participants with impaired fasting glucose may have resulted in lower effectiveness. More vigorous intervention strategies (e.g., more strategies to address psychological distress and negative family support) may be required to augment effectiveness among individuals with impaired fasting glucose.

There are several important limitations to note. First, the study population represented the heterogenous Indigenous population of the local area, primarily with Indigenous ancestry from the US and Mexico. While this was important to the community, it also resulted in a potential limitation. There are significant demographic, social, and behavioral differences between those who report Indigenous ancestry from these two regions that may moderate intervention effectiveness. While the effect modification analyses did not demonstrate differential effectiveness, it is possible that the sample size was too small to detect significant differences if they existed. Second, while the sample size accounted for attrition, we may not have had sufficient power to detect a difference due to loss to follow-up. The study staff implemented numerous strategies to enhance retention, yet 14% of the standard and 16% of the enhanced participants were not able to provide data at the 12-month time point.

## Conclusions

In conclusion, this study shows that adding strategies to address historical trauma to a culturally tailored diabetes prevention intervention was not more effective than the culturally tailored intervention alone. Explanations for these findings relate to the closely aligned design of the two interventions, the impact of the enhancements, as well as shortcomings in adherence. However, across both interventions, participants who attended more sessions lost more weight, underscoring the importance of intervention adherence.

## Data Availability

The dataset used in this study are available from the corresponding author if approved by the American Indian Community Action Board.

## Abbreviations

AIAN: American Indian and Alaskan Native
AICAB: American Indian Community Action Board
BMI: Body mass index
CES-D: Center for Epidemiological Studies-Depression
CI: Confidence interval
DPP: Diabetes Prevention Program
PTSD: Posttraumatic stress disorder
SD: Standard deviation
SDPI: Special Diabetes Program for Indians
